# Tracking and Characterization of Spinal Cord-Injured Patients by Means of RGB-D Sensors

**DOI:** 10.3390/s20216273

**Published:** 2020-11-04

**Authors:** Filippo Colombo Zefinetti, Andrea Vitali, Daniele Regazzoni, Caterina Rizzi, Guido Molinero

**Affiliations:** 1 Department of Management, Information and Production Engineering, University of Bergamo, 24044 Dalmine (BG), Italy; filippo.colombozefinetti@unibg.it (F.C.Z.); daniele.regazzoni@unibg.it (D.R.); caterina.rizzi@unibg.it (C.R.); 2 Azienda Socio Sanitaria Territoriale (ASST) Papa Giovanni XXIII, 24127 Bergamo, Italy; gmolinero@asst-pg23.it

**Keywords:** markerless motion capture, automatic pushing analysis, SCI patients, RGB-D sensors

## Abstract

In physical rehabilitation, motion capture solutions are well-known but not as widespread as they could be. The main limit to their diffusion is not related to cost or usability but to the fact that the data generated when tracking a person must be elaborated according to the specific context and aim. This paper proposes a solution including customized motion capture and data elaboration with the aim of supporting medical personnel in the assessment of spinal cord-injured (SCI) patients using a wheelchair. The configuration of the full-body motion capturing system is based on an asymmetric 3 Microsoft Kinect v2 sensor layout that provides a path of up to 6 m, which is required to properly track the wheelchair. Data elaboration is focused on the automatic recognition of the pushing cycles and on plotting any kinematic parameter that may be interesting in the assessment. Five movements have been considered to evaluate the wheelchair propulsion: the humeral elevation, the horizontal abduction of the humerus, the humeral rotation, the elbow flexion and the trunk extension along the sagittal plane. More than 60 volunteers with a spinal cord injury were enrolled for testing the solution. To evaluate the reliability of the data computed with SCI APPlication (APP) for the pushing cycle analysis, the patients were subdivided in four groups according to the level of the spinal cord injury (i.e., high paraplegia, low paraplegia, C7 tetraplegia and C6 tetraplegia). For each group, the average value and the standard deviation were computed and a comparison with similar acquisitions performed with a high-end solution is shown. The measurements computed by the SCI-APP show a good reliability for analyzing the movements of SCI patients’ propulsion wheelchair.

## 1. Introduction

Spinal cord injury is caused by one or more lesions of the spinal cord. Inside the spinal cord, there is a complex network of neural and vascular connections which can be affected by pathologies and traumatic injuries. In particular, 50% of them are due to road accident, 24% to fall, 17% to other causes, 6% to sports and 3% to extreme sports [1]. The most important classification of spinal cord injury is the International Standard for the Neurological Classification of SCI (ISNCSCI) created by the American Spinal Injury Association (ASIA) [2]. This classification permits us to evaluate the type of injury and the rehabilitation program in order to improve patients’ quality of life. Lesions are grouped into levels according to their position along the spinal cord, and each level is correlated to a specific loss of functionality. If the subject loses motor and/or sensitive functions because of a lesion of the spinal cord in the cervical segment, the patient’s disability is called tetraplegia. The disability is called paraplegia if the spinal cord-affected areas are the thoracic, lumbar, or sacral segments. When a person is subjected to a spinal cord injury, the wheelchair is the basic support to start the rehabilitation process and recover personal autonomy. The first step of the process with a wheelchair requires a setup made according to the patient’s stature and conditions (e.g., muscular force, comorbidities or other impairments). The setup can be also changed according to the level of ability of the user who is learning how to sit and act on the handrims. For the posture analysis and propulsion evaluation, the assessment is still based on direct and qualitative observation, with no standard instruments to measure the improvements in wheelchair use. The observational approach has some limits; in fact, it is subjective and depends on physicians’ and physiotherapists’ experience. Moreover, data are not stored and only evaluation related to functional scales is recorded. Thus, even if the medical personnel are qualified, a patient’s medical history results may be incomplete and accurate monitoring over time is almost impossible.

To overcome these drawbacks, digital technologies for tracking patient’s movements can be adopted. Motion capture (Mocap) systems have the potential to track and record real human motion. They allow recognizing, reproducing and analyzing the motion of specific districts or of the entire body. The kinematic data of any action performed during a rehabilitation process can be the basis of an improved way to assess a patient’s condition and monitor his/her evolution in the long term. However, Mocap systems require specific technological skills that are challenging for medical personnel, who prefer to continue with the standard and traditional approach based on visual assessment instead of investing resources to introduce new technologies.

The aim of this paper is the creation of a novel procedure to analyze the motion of spinal cord-injured patients using a wheelchair by means of a motion capture system based on inexpensive sensors that is easily usable by physicians and physiotherapists. The design of the procedure has been defined in collaboration with the medical staff of the hospital ASST Papa Giovanni XXIII in Bergamo, which is one of the most important public hospital in Italy. A software application has been developed to automatically execute the pushing cycle analysis and find the key measurements of specific movements which are useful in assessing the patient’s performance in a simple way and supporting the medical personnel with objective data during the decision process. The application allows physicians to collect medical information during the patient’s rehabilitation process.

For evaluating the reliability of the proposed solution, the research work compares the acquired motion data with those obtained with a professional high-level Mocap solution found in the literature.

In the first part, this paper introduces the state of the art relative to the use of innovative technologies as a new frontier for medical rehabilitation; then, the methodology for developing the whole procedure based on the use of a low-cost Mocap system is described. Finally, the outcomes are discussed and a quantitative analysis is presented comparing the results reached so far with the data available in the literature.

## 2. State of the Art

The analysis of the propulsion of a wheelchair allows us to assess the correctness of the upper limb movements and loads in spinal cord-injured patients. Actually, the measurement of the upper limb, shoulder and back performance may prevent the risk of patient injury caused by a wrong use of a wheelchair. The correct approach for safe and energy-saving pushing is based on the kinematic data of patient’s movements and the forces generated during the propulsion.

Nowadays, rehabilitation centers adopt observational methods to assess the propulsion of a wheelchair. In the scientific literature, several standard protocols have been defined to obtain assessment methods that are as objective as possible for both intra-operator and inter-operator. For example, Gowran et al. [3] propose a list of existing visual standard protocols for the evaluation of wheelchair propulsion. The claimed aim is to find key factors supporting the physiotherapists in defining the best setting of the wheelchair, preventing injury and postural diseases. However, this approach is mainly static and does not consider the movements of patients during the use of the wheelchair. Smith et al. [4] define a specific method, the Wheelchair Propulsion Test (WPT), which relies on the observation of a patient using the wheelchair along a straight path that is 10 m long. The data acquired are the time required, the number of pushes, the identification of the pushing style and a pushing cycle analysis. Even if WPT guarantees an objective approach, still no data about the patient’s movements are considered. Other research works have developed the assessment protocol, Wheelchair Skills Test (WST). This defines a set of tasks that the patient has to perform with the wheelchair while one or more physiotherapists visually assess the way the patient performs pushing cycles according to capacity, confidence, performance and training goal. In some research works, such as Lindquist et al. [5] and Askari et. al. [6], physiotherapists use video recordings of the patients’ performances for consulting previous assessments. Additionally, WST allows an objective inter-operator and intra-operator approach, but no patient movements are directly measured.

Other research works combine the use of video recording with biomedical signals of muscles using electromyography. The aim is to study what happens to the upper arms during wheelchair propulsion. In particular, Chow et al. [7] use electromyography with recorded video during wheelchair propulsion on a sloped ramp. The result is the correlation of muscular activity of the upper limb with each phase of the pushing cycle. Additionally, in this case no kinematic data are available.

With the aim of detecting and recording the movements and forces generated by the patients during wheelchair usage, innovative technologies have been introduced as tools for assessing the wheelchair propulsion by considering patients’ performance.

Some researchers have exploited sensorized wheelchairs. For example, the commercial solution Smartwheel [8] allows measuring the forces and torsions on a wheelchair directly from the wheels. While forces are acquired on the wheels, patients’ kinematic data are monitored adding a motion capture (Mocap) system [9,10].

A Mocap system tracks and recognizes the human body shape to reconstruct a virtual skeleton that follows the recorded movements. The virtual skeleton allows directly evaluating the kinematic data of the movement and correlating them to the medical assessment. Three main types of Mocap system can be used: inertial systems and optical marker-based or markerless solutions.

Inertial Mocap systems are based on inertial measurement units (IMUs) applied along the human body to record movements during wheelchair propulsion. Leving et al. [11] show how it is possible to track and record upper limb movements by applying IMUs on patient’s shoulders, elbows and wrists. This technology is able to register the position, velocity and acceleration of each IMU sensor; however, no data are directly available that describe the kinematic behaviors of the whole patient’s body on the wheelchair. Similarly, Slikke et al. [12,13] show the high accuracy of an IMU motion acquisition system for assessing the wheelchair propulsion of SCI patients in sports. Additionally, in this case data only correlate to a single point along the upper arms. Moreover, IMU sensors can be considered slightly invasive from the patients’ point of view, and the eventual slipping of the sensors along the arms during acquisition may affect the final results.

Marker-based Mocap systems are usually expensive but they guarantee a high accuracy and precision. For example, Boninger et al. [14] used the Optitrack system [15] to analyze the kinematics of the upper limb and Smartwheel to measure the forces generated during wheelchair propulsion; this is performed on a propulsion platform in order to execute a large number of pushing cycles in a room. The achieved results show that the main causes of injuries are due to the asymmetry between the right and left upper arm during the pushing cycles. Vegter et al. [10] used a marker-based Mocap system and a sensorized wheelchair for evaluating the mechanical efficiency and the overload of the shoulder articulation. The evaluation takes place during the first part of the rehabilitation process, during which patients learn how to use the wheelchair. The results demonstrate how the kinematic data permit one to optimize the symmetry of the propulsion to reduce the overload on the shoulder and prevent inflammation for a long-lasting period after the initial rehabilitation. Due to their high accuracy and precision, marker-based Mocap systems are usually considered as a reference by which to evaluate other Mocap solutions. Newsam et al. [16] exploited a Vicon system to analyze how SCI patients perform the propulsion step. In particular, the aim was to evaluate a set of specific parameters of arm movements during the pushing cycles to investigate the main differences in performance among patient groups with different levels of spinal cord injury. Eight movements were measured: humeral elevation, horizontal abduction of the humeral, humeral rotation, elbow flexion, forearm pronation, trunk extension, wrist flexion and wrist ulnar deviation. They have been measured in degrees at five specific steps of the pushing cycle: the initial contact of the hand on the pushrim, the hand on the top center of the wheel, the hand off the pushrim, the end of the follow through and the end of the arm return. The results reached can be used as a reference to validate similar research works.

The use of this technology is costly, time-consuming and requires a dedicate room; thus, it is not so widespread in rehabilitation centers.

Markerless Mocap systems instead exploit a set of low-cost portable sensors. The most diffused solutions in the literature adopt RGB-D sensors that combine a standard RGB video with the depth data of the scene. Rammer et al. [17] propose a propulsion platform on which children on a wheelchair perform many pushing cycles. The patient’s movements on the wheelchair are acquired by a system composed of three Microsoft Kinect v2 sensors positioned around the wheelchair. The system can detect kinematic metrics relevant in manual wheelchair propulsion. The obtained data are relevant but hardly usable by a medical staff because they are not directly usable for pushing cycle analysis. Furthermore, even if using a platform reduces the volume of acquisition, which is positive, it also obliges the wheels to move on straight binaries, and the pushing movements may be distorted.

Literature contributions are generally focused on the technical feasibility while neglecting the final users’ requirements and background. Actually, physicians and physiotherapists must be able at least to set up the hardware devices to track the patients and obtain results in a readable format with a few simple steps. In the last five years, the authors have designed a solution that is patient- and physician-driven to perform pushing cycle analysis by means of a low-cost Mocap system based on three Microsoft Kinect v2 devices. The layout of the Mocap system was optimized to be easily usable, fast to mount and easily replicable in a rehabilitation center [18,19]. Furthermore, a specific application was developed to automatically extrapolate from the acquired data the motion parameters needed to assess the pushing cycle [20].

Concerning the performance of markerless Mocap systems, several research works have compared them with professional marker-based systems. Galna et al. [21] compared the use of a single Microsoft Kinect v1 with the Vicon system [22] to track movements of the upper limbs of patients with Parkinson’s disease. The results demonstrate that the Kinect device is an adequate system for acquiring wide movements of the arms with a good accuracy (e.g., abduction, adduction and rotation). Instead, it is not suitable to acquire fine movements of the hands, such as hand clasping. Concerning the use of a wheelchair, Milgrom et al. [23] compared a markerless Mocap system composed of the Microsoft Kinect device with a software development kit (SDK) [24] to acquire the wheelchair propulsion executed by patients on a platform. The results highlight the good accuracy obtained with a Kinect-based Mocap system which is suitable for measuring the key features of pushing cycles.

## 3. Method

In order to define a technological solution applicable in the medical field, a methodology has been developed by the authors and refined through many years of experience [19]. First, a preliminary study allowed the design of a Mocap layout with 3 Microsoft Kinect v2 sensors to assess the wheelchair propulsion of an SCI patient along a straight path approximatively 6 m long [18]. Then, by involving healthy people, the kinematic data were analyzed in collaboration with medical personnel in order to find a correlation between the movements and the patients’ assessment. Starting from medical information described in [19], the goal of the SCI patient analysis was to simplify and organize the acquired biomechanical data in order to make available to medical staff only the information needed for the patient’s rehabilitation.

**Figure 1:**
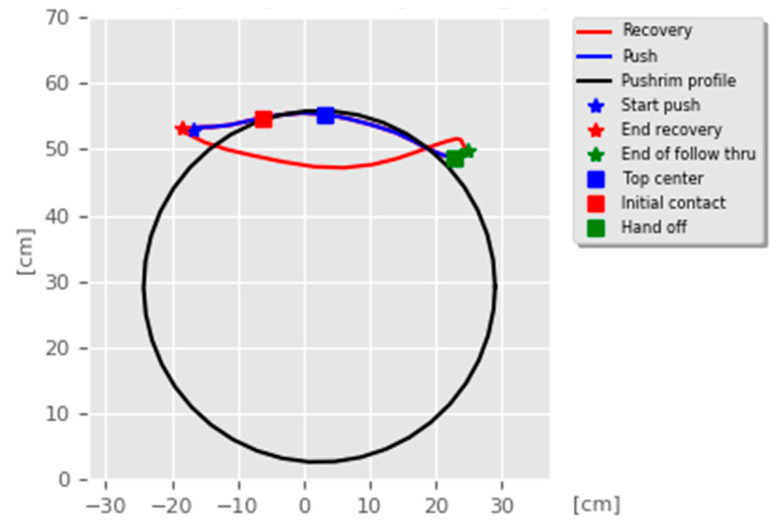
Example of a pushing cycle highlighting the recovery (red line) and push (blue line) movements as well as the considered key points.

A pushing cycle is subdivided in two main movements of the arms (Figure 1):

- Push: this occurs when the hands grab the wheelchair handrims and move the wheels forward.
- Recovery: this starts when the hands let the handrims go and finishes when the position of pushing is reached.

The beginning and the end of the push and recovery are very important because they correspond to the extreme reachable positions of the hands and determine the quality and effectiveness of the movements. According to the virtual skeleton describing the human, the hand joint corresponds to the real human wrist. It is connected to the forearm joint that is connected, in turn, to the shoulder joint. This kinematic chain, composed of only two links, is essential for the description of the hand propulsion trajectory. According to the physicians’ needs, an optimal pushing cycle analysis can be assessed if the patients can be tracked on a straight path at least 6 m long. Along the propulsion cycle, there are five key points (see Figure 1) that are used to compare the proposed solution with the data available in the literature:

- Top center (TC): the hand reaches the top center position of the pushrim during the push.
- Initial contact (IC): the hand touches the pushrim to start the push.
- Hand off (HO): the hand leaves the pushrim and then the push finishes.
- End of follow through (End FT): the hand finishes going forward before starting the recovery movement.
- End of arm return (End AR): the recovery movement finishes before starting a new pushing cycle.

Table 1 reports the measurements relative to the specific positions of arms, shoulders, and trunk useful for the assessment and also used for comparison with the results found in the literature. Each posture is described by the specific angles that are also shown graphically to permit an easy description and define the orientation.

**Figure 2:**
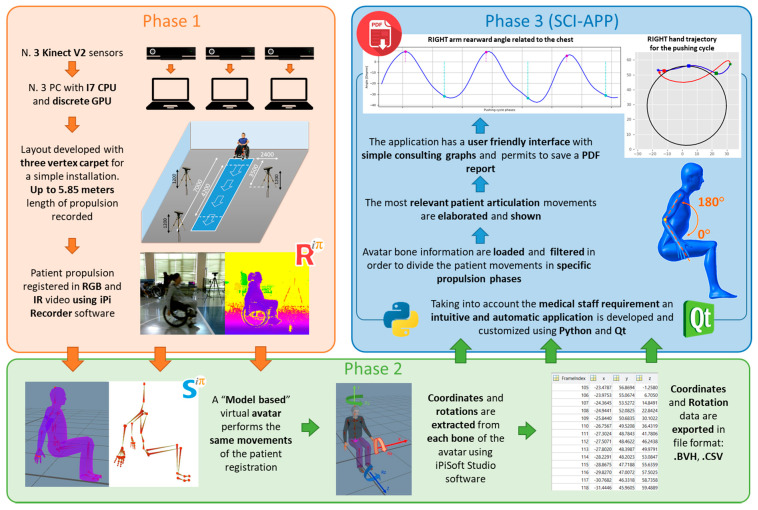
Main technological phases of the developed solution.

Figure 2 shows the main phases of the implemented solution. The first is relative to the design of the Mocap procedure to acquire the patient on the wheelchair along the straight path. The second phase is relative to the use of iPiSoft to elaborate the motion-captured data according to the movements useful for the pushing cycles analysis and the final assessment. The third phase is focused on the application named the Spinal Cord Injury APPlication (SCI-APP) that automatically analyzes the acquired data and generates information to support the medical personnel’s decisions.

### 3.1. Phase 1: Mocap Acquisition

According to the results reached in previous research works [18,20], the default settings have been modified to improve the maximum performance and give better frame rate parameters. A commercial software solution developed by iPi Soft [25] is used to record the human motion and to extract avatar joint information. The tracked movements and relative skeleton animation have been stored in BVH format.

According to the requirements of the medical staff and the technological limits of the adopted solution, the standard multiple depth sensor configuration of the Mocap software needs to be modified to allow a 5.85 m-long acquisition track and to record three complete propulsion cycles for almost any patient.

The Mocap acquisition was designed in order to establish a checklist to reduce accidental oversights and human errors. In this way, all the patient’s actions are taken with the same environmental conditions, hardware setup and software performance. This protocol was developed thanks to the experience gathered with a number of tests at the university laboratory and acquisition sessions. Table 2 describes the sequence of actions grouped according to the three main steps. The first foresees the layout assembly and all the PC connections, the second includes all the actions needed to calibrate the Mocap system and the last concerns how to use iPiSoft recorder.

Further documentation, not reported in this paper, was created to help users in managing files (e.g., about calibration or wheelchair specifications), to check that the lights and patient’s clothes are suitable and to properly inform patients about the procedure.

### 3.2. Phase 2: Data Extraction

This phase has the goal to extract from the acquired data the set of information required by physicians to evaluate the patient and his/her rehabilitation process. A set of correlations was defined between the raw data and the medical evaluation parameters. The adopted human avatar is composed of 29 joints with their associated positions, velocities and accelerations. The evaluated movements are associated with the kinematic data of the virtual joints. Table 3 depicts how medical parameters are quantified according to the tracked data. For the sake of the evaluation, it is also relevant to determine the entire trajectory of the hands during the pushing cycle. The beginning and end of any cycle are automatically identified by the ad hoc developed application. Furthermore, the movements are automatically measured according to the key points of each detected pushing cycle in order to obtain a complete analysis.

### 3.3. Phase 3: SCI-APP

By starting from the BVH file exported by iPisoft, SCI-APP automatically performs the analysis of the pushing cycles executed by the patients and makes available both linear and angular measurements of the upper limbs. All the information can be automatically exported in PDF and Microsoft Excel file format.

The whole application was developed in Python and is composed of four main modules:

- **Computation of pushing analysis.** This requires as input the kinematic data of the virtual skeleton to automatically detect all the instants relative to the beginning of both phases of the pushing cycle. It automatically checks if there are asymmetries between the left and the right pushing cycles.
- **Data extrapolation of each pushing cycles.** This makes available all the data of a specific pushing cycle. It is used to generate the graphs needed to compare the position of the hand with the position of the handrim and visually evaluates each type of pushing cycle.
- **Measurements of specific patient movements.** This computes the measurements of the human body articulations according to the medical reference system. The computed data are the linear and angular measurements described in Table 3.
- **Generation of the medical reports.** This permits us to automatically generate a report and save the computed data and measurements in a PDF or Excel file.

**Figure 3:**
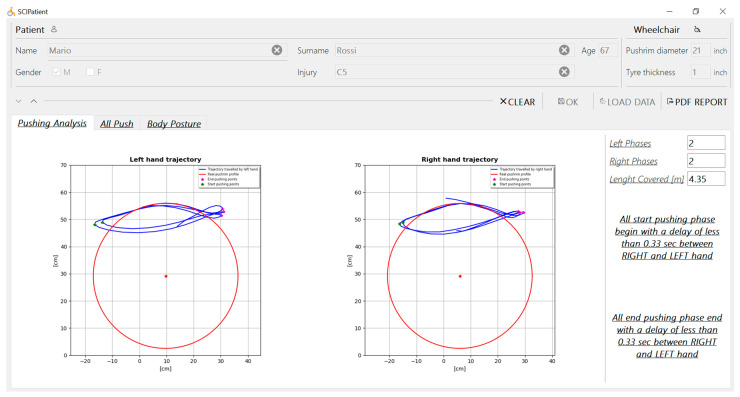
User interface of SCI-APP with an example of pushing analysis with the left and right hand trajectories with an asymmetry warning on the right side.

The medical personnel can access all these data through a user-friendly interface developed by means of Qt [26]. Figure 3 shows the SCI-APP user interface. The upper part is dedicated to the patients’ data that have to be filled in by the medical personnel. The bottom part includes three main tabs related to the main system functionalities, each with its graphical area, to show the computed outcomes.

The first tab, named “Pushing Analysis”, contains the data computed using the first module related to the propulsion analysis and its relative key points. In particular, the following information is shown:

- Number of right and left pushing phases made by the patient during wheelchair propulsion.
- Length covered by the patient during the wheelchair propulsion.
- A warning when the application detects symmetry loss during one or more propulsion phases.

Furthermore, it portrays two graphs about the right and left hand trajectory according to the position of the handrim profile (Figure 3). The beginning and the end of the detected pushing phases are highlighted for both the right and left sides. In this way, it is possible to show the extreme positions of upper limbs relative to the handrim during propulsion.

**Figure 4:**
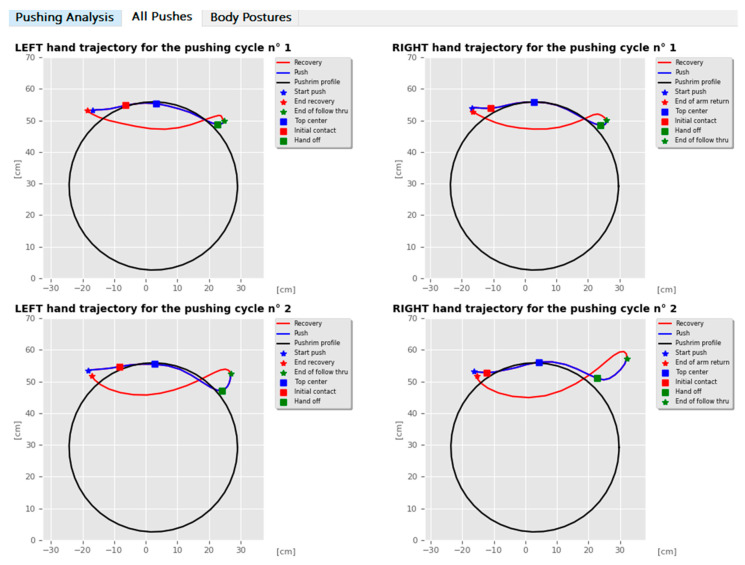
Examples of a trajectory analysis of two pushing cycles for both the left and right hands automatically calculated by SCI-APP.

The second tab, entitled “View All Pushes”, exploits the second module and shows the graphs of each right and left pushing cycle. Supported by these data, the operator can easily classify each pushing cycle according to the performed trajectory and easily find the difference between the right and left hand gesture during each propulsion movement (Figure 4).

**Figure 5:**
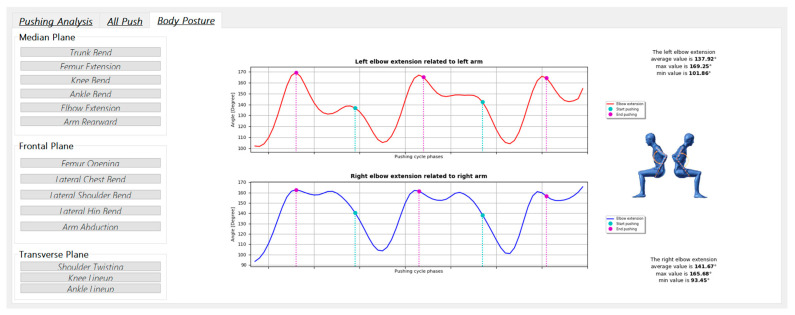
Example of an “Elbow flection” analysis with two graphs related to the specific movements measured according to the median, frontal and transverse planes and a graphical representation.

**Figure 6:**
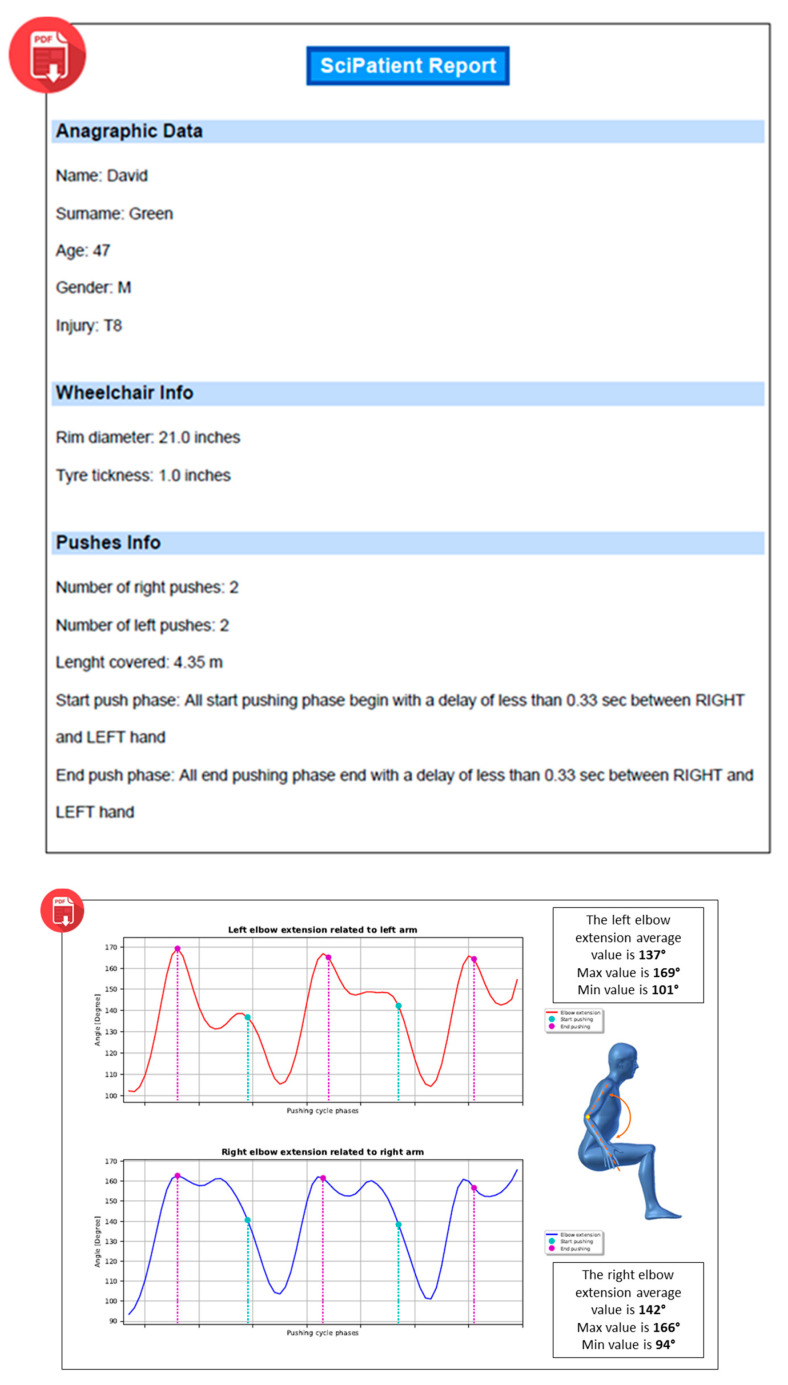
An example of a PDF report generated by SCI-APP including the main patient’s data, information about the acquired pushing cycles (upper part) and the results of the analysis with graphical representations (lower part).

The third tab, named “Body Posture”, makes available graphs plotting the key parameters that describe the patient’s motion (Table 2) for the entire acquisition. Furthermore, each graph shows the minimum, maximum and average values of relative parameters; the start and end pushing frames are highlighted in different colors to correlate the pushing cycle phases with the behavior of the evaluated movement (Figure 5). Finally, the operator can generate a pdf report with all the information needed for the medical assessment, as shown in Figure 6.

In order to evaluate the reliability of the data and the measurements computed with SCI-APP, a comparison must be carried out with similar acquisitions performed with a high-end solution. In this research study, the results of Newsam et al. [16] were chosen as reference data for the comparison. SCI-APP exports the useful data in Excel file format for each acquisition. In particular, for each pushing cycle the 5 key points shown in Figure 1 are computed and for each of them are shown the measurements of the 5 movements to evaluate the wheelchair propulsion: the humeral elevation, the horizontal abduction of the humerus, the humeral rotation, the elbow flexion and the trunk extension along the sagittal plane. These movements are measured in degrees using the kinematic data of the virtual joints, as depicted in Table 3.

The goal is to investigate if low-cost sensors can track the wheelchair propulsion with an accuracy that can be considered from good to excellent. According to [21,22,23,24,25,26,27], a markerless Mocap system can be considered good for the evaluation of medical parameters relative to the movements if the measured movement has either an overestimation or underestimation reasonably smaller than 10°, and excellent when either the overestimation or underestimation is reasonably smaller than 5°.

## 4. Campaign of Acquisition

The acquisition of the SCI patients was performed at the rehabilitation center of the Hospital ASST Papa Giovanni XXIII, the main public hospital in Bergamo, Italy. Sixty volunteers with spinal cord injury were recorded. They were divided in four groups according to the level of the injury lesion: high paraplegia (*n* = 15 patients), low paraplegia (*n* = 15 patients), C6 tetraplegia (*n* = 13 patients) and C7 tetraplegia (*n* = 15 patients). Concerning gender, 80% of the patients were male and 20% female.

**Figure 7:**
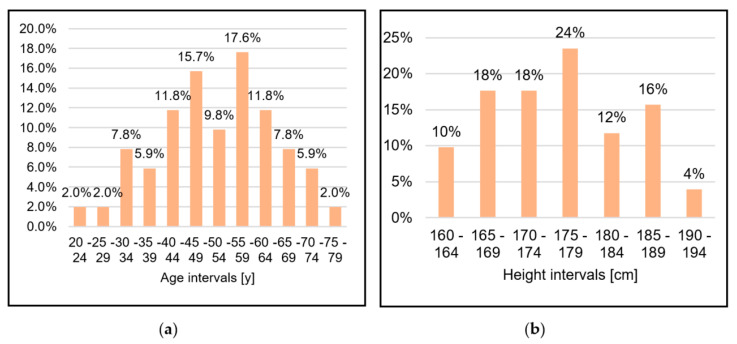
Distribution of patients by age (**a**) and height (**b**).

**Figure 8:**
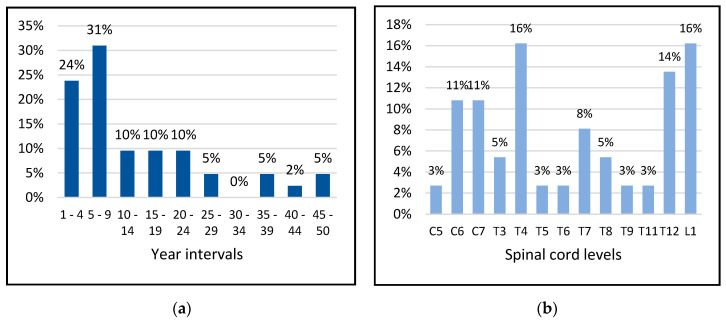
Distribution of patients by how many years since the injury occurred (**a**) and height of the spine lesion (**b**).

Figure 7 shows the distribution of the patients by age and height, and Figure 8 shows the distribution of the patients by how many years since the injury occurred and the height of the spinal lesion.

The exclusion criteria are centered on the functional evaluation of patients; age, sex, height or kind of lesion do not limit the patients from being enrolled, but only their ability to use a wheelchair.

**Figure 9:**
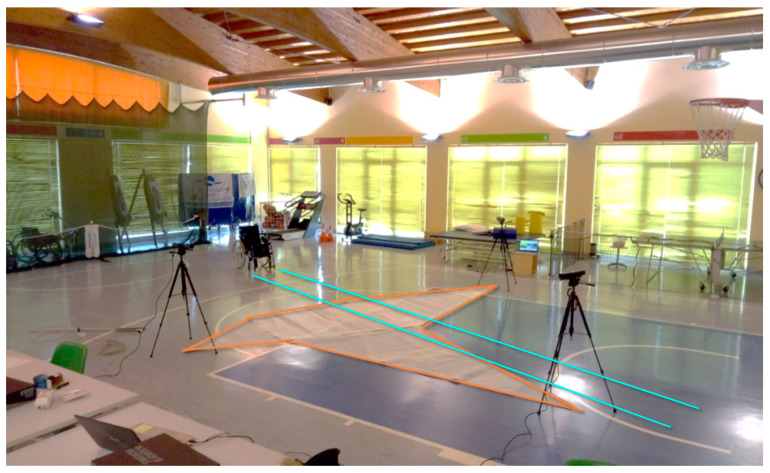
Rehabilitation gym and sensors layout with three-vertex carpet and wheelchair path. This tool allows a faster preparation of the layout of the Mocap system and a reference surface to correctly calibrate the multiple Microsoft Kinect sensors.

Figure 9 shows the rehabilitation gym of the hospital where the experiment was carried out. To avoid variation in the light during long test sessions, artificial lights were preferred to natural light. The three Kinect V2 sensors were placed according to the defined layout using the positioning carpet. Each vertex of the carpet corresponds to the position of a sensor.

**Figure 10:**
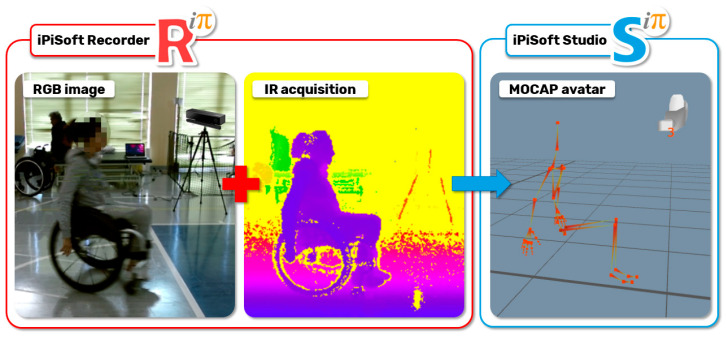
Respectively, RGB image, IR acquisitions and the virtual avatar of the acquired patient.

Patients are asked to practice on the path before being tracked. Each patient was acquired twice. The generated virtual avatar was exported by iPiSoft in a BVH file (Figure 10), which was used to automatically perform the pushing analysis with the SCI-APP and export the patient’s body motions (Table 3) at each key position (i.e., IC, TC, HO, end FT and end AR).

In total, 138 acquisitions have been accomplished. Even if patients have been trained and informed to wear tight clothes, it happened that some acquisitions failed when the data were processed. Unfortunately, it is not possible to check all the parameters in real time and, thus, some acquisitions may fail. Anyway, this could be easily solved if the procedure becomes a routine activity in a rehabilitation center. A total of 116 acquisitions out of 138 (85%) are correct and have been automatically processed by the SCI-APP application.

The average duration of the video acquisition was 105 frames, corresponding to 3.5 s at 30 fps; eventual differences due to slow movements or other conditions can be easily handled. For each acquisition, the SCI-APP application is able to identify, from an average of 4.30 m net acquisition path length, two complete cycles (51%) or three complete cycles (24%). The number of single pushes (i.e., without recovery) goes from two to five in 96% of the cases analyzed. In 9% of the acquisitions analyzed, the SCI-APP detected asymmetric movements between the right and left upper limbs, which involves a different number of pushing phases between the right and the left hands.

There are some drawbacks regarding the use of an optical system to track part of the body—e.g., the pelvis and hips—because they are occluded by the presence of the wheelchair even if these parts are not crucial for patients’ assessment.

## 5. Results and Discussion

In the next subsections, the results reached so far are presented and discussed as follows. For each patient group, the average value and the standard deviation have been computed for each of the five considered movements in each key point and compared with data reported in Newsam et al. [16].

A table is presented for each movement. The table has three rows for each patient group; the first reports the reference data (i.e., the data reported in Newsam et al. [16]), the data computed by the SCI APP and the difference between them. The columns are relative to the five key points of the pushing cycle.

### 5.1. Humeral Elevation

Table 4 shows the parameters relative to the humeral elevation. The ***Δ*** rows show average values very good compared to the reference values for all the key instants in all the groups of patients. In detail, IC, TC and end AR present an optimal measurement using Kinect sensors. This result confirms that the Kinect sensors can track wide movements of the human body as the humeral elevation with a high quality [28,29].

The key points HO and the end FT have the highest ***Δ***. The difference could be due to the fact that Newsam et al. performed the acquisition with a shorter path of 4 m. This may affect the way the patient pushes the wheelchair propulsions [28,29].

### 5.2. Horizontal Abduction of Humerus

Table 5 shows the parameters relative to the horizontal abduction of the humerus. The ***Δ*** rows show the average values optimal for the key points IC, TC, end FT and end AR for all the groups of patients. Additionally, in this case the high similarity confirms the potentialities of Kinect sensors to track wide movements.

The key points HO has a very high ***Δ*** value. Additionally in this case, the difference could reasonably depend on the length of the acquired path.

### 5.3. Humeral Rotation

Table 6 shows the parameters relative to the humeral rotation. The ***Δ*** rows show average values that are very good for the key points IC, TC and end AR for all groups of patients. Very high ***Δ*** values have been computed for HO and end FT. In this case, it is very difficult to understand if the high difference can be correlated with a lower propulsion or if there is a loss of the correct humeral rotation by the Mocap system during tracking in the second part of the propulsion. The loss of tracking may be caused by a type of movement (i.e., the rotation of the humerus around own axis) that can be very hard to detect using optical sensors.

### 5.4. Elbow Flexion

The evaluation of the elbow flexion/extension is shown in Table 7. The ***Δ*** rows show average values good for the key points IC, TC, end FT and end AR for all the groups of patients. There are higher ***Δ*** values only for the group with low paraplegia, but the values of their standard deviation permit us to consider the differences negligible. The possibility of shorter propulsions is further confirmed by the ***Δ*** values of HO of the elbow flexion/extension. Additionally in this case, the HO values are higher than the reference data and the reason for this could be the same as for the previous cases.

### 5.5. Trunk Flexion/Extension

The trunk flexion/extension along the sagittal plane is very similar for each group of patients. In this case, the data from the literature [16] make available a general average value for all the key instants that have a value between 1 and 7 degrees. According to this range, the values measured with the SCI-APP are very good (Table 8). Furthermore, Newsam et al. highlighted that the subjects with C6 tetraplegia have a total excursion of trunk motion significantly greater than all the other groups (i.e., an average value of 11°), which is confirmed by the data we acquired.

### 5.6. Final Considerations

Concerning the information available in the scientific literature [16,21,27,28,29], the outcomes reached with the SCI-APP are consistent. Moreover, the long path we are using allows tracking more pushing cycles for each acquisition, making the average values more robust. Actually, tracking patients on a short path, even with top-quality sensors, can influence the way patients behave. This condition may be one of the reasons why the values of movements at key points HO and end FT are not similar to those available in the literature.

The comparison performed so far has been shown to the involved medical personnel and their feedback is positive. Thus, we are confident that the presented markerless Mocap system can be easily adopted as a tool for the medical assessment of pushing cycles.

Further investigations have been planned to analyze in detail the movement of the humerus, since it is the most relevant contribution to the entire pushing. The main weakness of the presented solution seems to be the measurements of the rotations along the axes of virtual bones, as described in § 5.3.

Future developments will be dedicated to tracking the movements of wrists, hands and fingers, which have been neglected in this research work.

## 6. Conclusions

Paraplegia is mainly due to injuries to the spinal cord and may cause paralysis of the lower limbs; the wheelchair is the best moving solution for most people with this disability. The assessment of posture and the way the wheelchair is used is essential during the rehabilitation process to avoid further ailments or excessive energy expenditure. A structured method has been provided to support the complex issue of evaluating the way a person moves on a wheelchair. The method has been used with 60 patients, whose data have been elaborated by an ad hoc developed application to obtain the required medical parameters. As a result, a simple but flexible user interface allows plotting any translation or rotation of the involved articulations that may be relevant for the assessment. Physiotherapists are provided with automatic alerts to highlight critical conditions, such as asymmetry of the movements, and reports are generated automatically to keep track of the outcomes. The medical knowledge collected and formalized allowed developing a solution that not only provides medical personnel with the exact information they need, but that is also suitable for easy use.

The data generated have been compared with the few references available in the literature, which use costly Mocap systems for tracking. The comparison was conducted on the key movements of the trunk and arms in five specific instants of the pushing cycle. These data were evaluated by subdividing the patients into four groups according to the level of spinal cord injury. The comparison has highlighted mean values very similar to those in the scientific literature. A systematic error for the parameters HO and end-FT was highlighted and the most probable cause is correlated with the procedure of acquisition, which required patients to execute a possible pushing cycle along a straight path. This approach generated a pushing cycle shorter than the natural performance. In general, the results reached confirm that SCI-APP and the proposed markerless solution are useful for an adequate evaluation of propulsion.

Further works will be dedicated to performing a usability test involving personnel who did not participate to the development of the solution in order to highlight the eventual limits. Moreover, a validation campaign will be carried out considering the possibility of using more precise and accurate sensors. After this, the proposed solution will be ready for broad use in rehabilitation centers.

## Figures and Tables

**Table 1:** Main movements characterizing a patient’s posture during wheelchair propulsion. The description is based on four pieces of information: the plane on which the movement is performed, the human articulation performing the movement, an image to visually understand how the movement is assessed and a description of the biomechanics of the movement considered.

Plane Considered	Human Articulation Requested	Position/Movement	Biomechanics Joint Analyzed
Median Plane	Trunk Flection	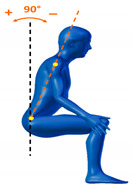	It contributes to determining the patient stability during wheelchair propulsion. As the angle decreases, the stability increases, but over a threshold angle the wheelchair can overturn during the pushing phase.
Median Plane	Humeral Elevation	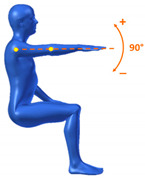	The humeral elevation describes the rotation of the shoulder along the median plane during the propulsion of the wheelchair.
Median Plane	Humeral Rotation	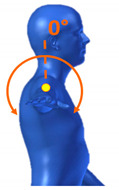	The humeral rotation is the rotation around the axes of the humerus.
Transverse Plane	Humeral Horizontal Abduction	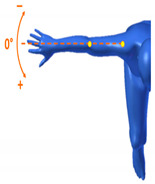	The horizontal abduction of the humerus describes the rotation of the shoulder around the transverse plane during the propulsion of the wheelchair.
Median Plane	Elbow Flexion/Extension	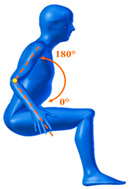	The maximum and minimum values of this angle have to stay in an optimum range in order to prevent a problem with the elbow articulation. This angle depends on the seat translation compared to the wheel rotation axle. Right and left angles are compared to assess the symmetric propulsion.

**Table 2:** The action checklist for a correct Mocap acquisition of the SCI patients.

To Do List for SCI Patients’ Acquisitions				
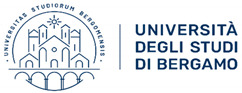				
LAYOUT	To repeat once a day		1.1	Lay out the three-vertex carpet
			1.2	Check Kinect 1.20 m in height
			1.3	Check Kinect horizontal inclination of −13°
			1.4	Check vertical orientation of Kinect RGB camera field of view
			1.5	Check ambient light source to darken (windows, lamps)
			1.6	Check Kinect - PC USB cable link
			1.7	Check ethernet cable link
			1.8	Setup iPi Soft Recorder Master and Slaves computers
			1.9	Setup new folder of the acquisition day “YYYY-MM-DD”
CALIBRATION 120 [sec]	To repeat for each calibration approx. every 30 min		2.1	Background iPi Soft Recorder 10 sec (with carpet on the floor), the Kinect field of view must be without anyone
			2.2	Setup Kinect with glass filter
			2.3	Spiral movements with light marker + Recording using iPi Soft Recorder
			2.4	Delete PC slave videos -> Button “Merge video” in iPi Soft Recorder
			2.5	Calibration using iPi Soft Studio
			2.6	Take off glass filter from Kinect
			2.7	Take off carpet from the floor
			2.8	Background iPi Soft Recorder 10 [sec] (without carpet on the floor), the Kinect field of view must be without anyone
VIDEO RECORDING	To repeat for each patient		3.1	Setup new patient’s folder “No. - Patient Surname”
			3.2	Change folder directory in iPi Soft Recorder
		To repeat for each acquisition	3.3	Registration using iPi Soft Recorder
			3.4	Delete PC slave videos -> Button “Merge video” in iPi Soft Recorder

**Table 3:** Correlations between human articulations and virtual joints. The left column reports the measured human articulations by considering a specific set of virtual joints highlighted in green in the images depicted in the right column. The central column describes how to measure the movements using the virtual joints.

Human Articulation	Data and Information		Virtual Joints and Segments
Elbow Flection	Joints	Lower spine, neck.	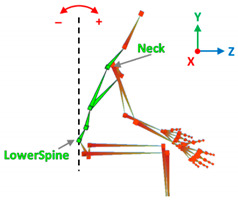
	Angle [°]	*X*-axis rotations.	
	Description	The bending of the trunk is measured as the angle between the vertical line and the segment passing between the lower spine and neck.	
Humeral Elevation	Joints	R/L shoulder, R/L forearm.	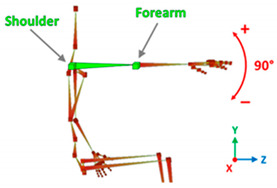
	Angle [°]	*X*-axis rotations.	
	Description	The humeral elevation is measured as the rotation of the shoulder joint around the *X*-axis.	
Humeral Rotation	Joints	R/L shoulder.	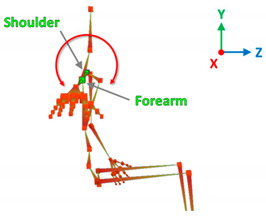
	Angle [°]	*X*-axis rotations.	
	Description	The humeral rotation is measured as the rotation of the shoulder joint around the axis defined as the vector between the shoulder position and the forearm position.	
Humeral Horizontal Abduction	Joints	R/L shoulder, R/L forearm.	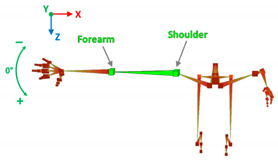
	Angle [°]	*Y*-axis rotations.	
	Description	The humeral horizontal abduction is measured as the rotation of the shoulder joint around the X-axis.	
Elbow Flexion/Extension	Joints	L/R forearm.	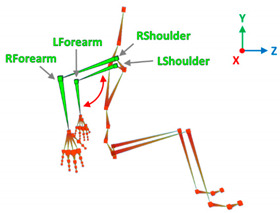
	Angle [°]	*X*-axis rotations.	
	Description	During the propulsion phase, the angles considered have periodic movements. Max and min extensions are significant data by which to assess the upper limbs’ performance.	

**Table 4:** Values of the humeral elevation in degrees for each group of patients.

*Humeral Elevation*	IC		TC		HO		End FT		End AR	
	Avg.	St.D.	Avg.	St.D.	Avg.	St.D.	Avg.	St.D.	Avg.	St.D.
Low paraplegic (Ref. Val.)	55.1	4.4	48.8	4.3	24.2	4.6	22.1	3.9	56.9	4.7
**Low paraplegic (Sci App)**	**52.0**	**7.3**	**46.7**	**8.2**	**16.7**	**15.1**	**11.8**	**6.5**	**54.3**	**9.1**
***Δ***	**−*3.1***		**−*2.1***		**−*7.5***		**−*10.3***		**−*2.6***	
High paraplegic (Ref. Val.)	53.8	7.8	47.1	7.9	23.7	4.3	22.1	4.0	55.7	7.2
**High paraplegic (Sci App)**	**51.3**	**8.7**	**45.7**	**10.4**	**17.2**	**14.7**	**10.0**	**8.7**	**50.5**	**10.2**
***Δ***	**−*2.5***		**−*1.4***		**−*6.5***		**−*12.1***		**−*5.2***	
C7-tetraplegic (Ref. Val.)	49.0	8.9	42.6	9.7	22.1	4.4	21.5	4.2	52.5	7.9
**C7-tetraplegic (Sci App)**	**44.8**	**6.9**	**37.7**	**6.8**	**7.8**	**6.6**	**10.9**	**8.5**	**43.4**	**8.9**
***Δ***	**−*4.2***		**−*4.9***		**−*14.3***		**−*10.6***		**−*9.1***	
C6-tetraplegic (Ref. Val.)	45.4	8.3	41.1	9.2	23.8	6.9	21.6	5.5	49.5	8.0
**C6-tetraplegic (Sci App)**	**41.3**	**6.8**	**38.6**	**5.3**	**15.0**	**9.4**	**13.8**	**5.7**	**40.8**	**5.5**
***Δ***	**−*4.1***		**−*2.5***		**−*8.8***		**−*7.8***		**−*8.7***	

**Table 5:** Values of the abduction of humerus in degrees for each group of patients.

*Horizontal Abduction*	IC		TC		HO		End FT		End AR	
	Avg.	St.D.	Avg.	St.D.	Avg.	St.D.	Avg.	St.D.	Avg.	St.D.
Low paraplegic (Ref. Val.)	−53.6	8.1	−41.8	8.5	6.8	12.5	21.8	14.8	−55.3	8.3
**Low paraplegic (Sci Lab)**	**−51.7**	**10.1**	**−46.7**	**10.8**	**−13.9**	**21.1**	**22.8**	**18.8**	**−51.2**	**10.2**
***Δ***	***1.9***		**−*4.9***		**−*20.7***		***1.0***		***4.1***	
High paraplegic (Ref. Val.)	−55.5	8.9	−44.8	6.8	0.8	20.7	15.6	24.0	−56.5	8.8
**High paraplegic (Sci Lab)**	**−54.2**	**9.1**	**−46.4**	**9.2**	**−18.2**	**18.1**	**15.1**	**18.4**	**−52.1**	**8.8**
***Δ***	***1.3***		**−*1.6***		**−*19.0***		**−*0.5***		***4.4***	
C7-tetraplegic (Ref. Val.)	−58.3	7.7	−48.8	6.8	8.6	17.9	19.6	21.1	−59.3	6.7
**C7-tetraplegic (Sci Lab)**	**−59.2**	**8.3**	**−52.8**	**10.2**	**−5.4**	**20.3**	**23.4**	**18.7**	**−57.0**	**6.1**
***Δ***	**−*0.9***		**−*4.0***		**−*14.0***		***3.8***		***2.3***	
C6-tetraplegic (Ref. Val.)	−54.6	8.4	−43.5	13.3	8.2	14.2	9.9	15.4	−55.1	8.4
**C6-tetraplegic (Sci Lab)**	**−56.2**	**8.3**	**−51.1**	**7.8**	**−9.9**	**29.3**	**17.9**	**11.0**	**−52.1**	**7.1**
***Δ***	**−*1.6***		**−*7.6***		**−*18.1***		***8.0***		***3.0***	

**Table 6:** Values of the humeral rotation in degrees for each group of patients.

*Humeral Rotation*	IC		TC		HO		End FT		End AR	
	Avg.	St.D.	Avg.	St.D.	Avg.	St.D.	Avg.	St.D.	Avg.	St.D.
Low paraplegic (Ref. Val.)	78.0	14.8	69.9	14.6	37.0	18.7	24.4	22.2	77.7	14.5
**Low paraplegic (Sci Lab)**	**74.0**	**3.6**	**70.6**	**4.1**	**53.1**	**10.4**	**36.6**	**10.3**	**75.0**	**5.0**
***Δ***	**−*4.0***		***0.7***		***16.1***		***12.2***		**−*2.7***	
High paraplegic (Ref. Val.)	75.6	14.5	67.8	14.4	35.5	26.4	22.4	31.6	76.0	14.2
**High paraplegic (Sci Lab)**	**75.7**	**4.6**	**71.8**	**4.3**	**57.1**	**8.5**	**43.2**	**7.0**	**75.5**	**5.0**
***Δ***	***0.1***		***4.0***		***21.6***		***20.8***		**−*0.5***	
C7-tetraplegic (Ref. Val.)	73.6	12.9	70.7	12.6	27.2	22.4	17.3	27.5	72.0	12.9
**C7-tetraplegic (Sci Lab)**	**68.7**	**6.0**	**63.5**	**5.2**	**43.0**	**9.3**	**34.6**	**7.1**	**67.6**	**5.8**
***Δ***	**−*4.9***		**−*7.2***		***15.8***		***17.3***		**−*4.4***	
C6-tetraplegic (Ref. Val.)	74.6	10.4	69.3	15.2	27.6	18.9	19.1	23.1	72.6	10.2
**C6-tetraplegic (Sci Lab)**	**70.6**	**5.0**	**68.1**	**5.2**	**52.1**	**10.6**	**37.5**	**8.4**	**70.3**	**3.4**
***Δ***	**−*4.0***		**−*1.2***		***24.5***		***18.4***		**−*2.3***	

**Table 7:** Values of the elbow flexion/extension in degrees for each group of patients.

*Elbow Flexion*	IC		TC		HO		End FT		End AR	
	Avg.	St.D.	Avg.	St.D.	Avg.	St.D.	Avg.	St.D.	Avg.	St.D
Low paraplegic (Ref. Val.)	59.4	10.9	76.4	8.9	43.1	9.8	34.8	9.0	54.3	9.8
**Low paraplegic (Sci Lab)**	**68.2**	**12.0**	**72.7**	**10.1**	**58.1**	**19.0**	**33.6**	**13.2**	**64.4**	**15.1**
***Δ***	***8.8***		**−*3.7***		***15.0***		**−*1.2***		***10.1***	
High paraplegic (Ref. Val.)	59.8	11.6	77.1	11.1	46.1	11.7	37.4	12.7	55.0	11.1
**High paraplegic (Sci Lab)**	**64.2**	**12.7**	**74.0**	**9.5**	**63.8**	**14.8**	**41.8**	**12.3**	**57.5**	**15.1**
***Δ***	***4.4***		**−*3.1***		***17.7***		***4.4***		***2.5***	
C7-tetraplegic (Ref. Val.)	65.5	8.1	77.1	8.1	42.4	10.1	35.4	10.1	62.9	9.2
**C7-tetraplegic (Sci Lab)**	**62.9**	**12.4**	**72.7**	**6.3**	**51.7**	**14.7**	**35.6**	**11.8**	**55.9**	**17.5**
***Δ***	**−*2.6***		**−*4.4***		***9.3***		***0.2***		**−*7.0***	
C6-tetraplegic (Ref. Val.)	62.8	10.0	69.8	10.7	42.8	9.3	41.8	9.3	61.3	10.6
**C6-tetraplegic (Sci Lab)**	**60.9**	**15.0**	**71.3**	**10.0**	**54.0**	**15.5**	**33.4**	**10.8**	**58.2**	**14.4**
***Δ***	**−*1.9***		***1.5***		***11.2***		**−*8.4***		**−*3.1***	

**Table 8:** Values of the trunk flexion/extension rotation in degrees for each group of patients.

*Trunk Extension*	IC		TC		HO		End FT		End AR	
	Avg.	St.D.	Avg.	St.D.	Avg.	St.D.	Avg.	St.D.	Avg.	St.D
Low paraplegic	−3.4	4.9	−3.8	5.0	−3.9	5.3	−2.6	5.2	−2.6	5.1
High paraplegic	0.0	7.7	0.0	7.8	0.1	6.8	0.9	6.3	0.3	7.1
C7-tetraplegic	0.7	9.4	0.0	9.1	−0.9	8.7	−0.9	8.8	0.9	8.9
C6-tetraplegic	12.4	5.1	11.0	5.0	9.1	5.1	9.6	5.5	12.4	5.4
